# Multicenter Case–Control Study of COVID-19–Associated Mucormycosis Outbreak, India

**DOI:** 10.3201/eid2901.220926

**Published:** 2023-01

**Authors:** Valliappan Muthu, Ritesh Agarwal, Shivaprakash Mandya Rudramurthy, Deepak Thangaraju, Manoj Radhakishan Shevkani, Atul K. Patel, Prakash Srinivas Shastri, Ashwini Tayade, Sudhir Bhandari, Vishwanath Gella, Jayanthi Savio, Surabhi Madan, Vinay Kumar Hallur, Venkata Nagarjuna Maturu, Arjun Srinivasan, Nandini Sethuraman, Raminder Pal Singh Sibia, Sanjay Pujari, Ravindra Mehta, Tanu Singhal, Puneet Saxena, Varsha Gupta, Vasant Nagvekar, Parikshit Prayag, Dharmesh Patel, Immaculata Xess, Pratik Savaj, Naresh Panda, Gayathri Devi Rajagopal, Riya Sandeep Parwani, Kamlesh Patel, Anuradha Deshmukh, Aruna Vyas, Srinivas Kishore Sistla, Priyadarshini A Padaki, Dharshni Ramar, Saurav Sarkar, Bharani Rachagulla, Pattabhiraman Vallandaramam, Krishna Prabha Premachandran, Sunil Pawar, Piyush Gugale, Pradeep Hosamani, Sunil Narayan Dutt, Satish Nair, Hariprasad Kalpakkam, Sanjiv Badhwar, Kiran Kumar Kompella, Nidhi Singla, Milind Navlakhe, Amrita Prayag, Gagandeep Singh, Poorvesh Dhakecha, Arunaloke Chakrabarti

**Affiliations:** Postgraduate Institute of Medical Education and Research, Chandigarh, India (V. Muthu, R. Agarwal, S.M. Rudramurthy, N. Panda, A. Chakrabarti);; Kovai Medical Center and Hospital, Coimbatore, India (D. Thangaraju, G.D. Rajagopal);; Avron Hospitals, Ahmedabad, India (M.R. Shevkani, R.S. Parwani);; Sterling Hospital, Ahmedabad (A.K. Patel, K. Patel);; Sir Gangaram Hospital, New Delhi, India (P.S. Shastri);; Kingsway Hospital, Nagpur, India (A. Tayade, A. Deshmukh);; Sawai Man Singh Medical College, Jaipur, India (S. Bhandari, A. Vyas);; Asian Institute of Gastroenterology, Hyderabad, India (V. Gella, S.K. Sistla);; St. John’s Medical College and Hospital, Bengaluru, India (J. Savio, P.A. Padaki);; Care Institute of Medical Sciences, Ahmedabad (S. Madan, D. Ramar);; All India Institute of Medical Science Bhubaneswar, Odisha, India (V.K. Hallur, S. Sarkar);; Yashoda Hospitals, Hyderabad (V.N. Maturu, B. Rachagulla);; Royal Care Hospital, Coimbatore (A. Srinivasan, P. Vallandaramam);; Apollo Hospitals, Chennai, India (N. Sethuraman, K.P. Premachandran);; Government Medical College, Patiala, India (R.P.S. Sibia, S. Pawar);; Poona Hospital and Research Centre, Pune, India (S. Pujari, P. Gugale);; Apollo Hospitals, Bengaluru (R. Mehta, H. Kalpakkam, P. Hosamani, S.N. Dutt, S. Nair);; Kokilaben Dhirubhai Ambani Hospital and Medical Research Institute, Mumbai, India (T. Singhal, S. Badhwar);; Army Hospital (Research and Referral), New Delhi (P. Saxena, K.K. Kompella);; Government Medical College, Chandigarh (V. Gupta, N. Singla);; Global Hospital, Mumbai (V. Nagvekar, M. Navlakhe);; Deenanath Mangeshkar Hospital, Pune (P. Prayag, A. Prayag);; City Clinic and Bhailal Amin General Hospital, Vadodara, India (D. Patel);; All India Institute of Medical Sciences, New Delhi (I. Xess, G. Singh);; Institute of Infectious Disease and Critical Care Hospital, Surat, India (P. Savaj, P. Dhakecha)

**Keywords:** Mucormycosis, Mucorales, COVID-19, Zygomycosis, invasive molds, coronavirus disease, Rhizopus, aspergillosis, fungi, SARS-CoV-2, viruses, zoonoses, respiratory infections, severe acute respiratory syndrome coronavirus 2, India

## Abstract

We performed a case–control study across 25 hospitals in India for the period of January–June 2021 to evaluate the reasons for an COVID-19–associated mucormycosis (CAM) outbreak. We investigated whether COVID-19 treatment practices (glucocorticoids, zinc, tocilizumab, and others) were associated with CAM. We included 1,733 cases of CAM and 3,911 age-matched COVID-19 controls. We found cumulative glucocorticoid dose (odds ratio [OR] 1.006, 95% CI 1.004–1.007) and zinc supplementation (OR 2.76, 95% CI 2.24–3.40), along with elevated C-reactive protein (OR 1.004, 95% CI 1.002–1.006), host factors (renal transplantation [OR 7.58, 95% CI 3.31–17.40], diabetes mellitus [OR 6.72, 95% CI 5.45–8.28], diabetic ketoacidosis during COVID-19 [OR 4.41, 95% CI 2.03–9.60]), and rural residence (OR 2.88, 95% CI 2.12–3.79), significantly associated with CAM. Mortality rate at 12 weeks was 32.2% (473/1,471). We emphasize the judicious use of COVID-19 therapies and optimal glycemic control to prevent CAM.

## Abstract 2

We found inappropriate glucocorticoid therapy and zinc supplementation were significantly associated with these illnesses.

Mucormycosis is an invasive fungal infection associated with high death rates. Poorly controlled diabetes mellitus, organ transplantation, hematological malignancies, and immunosuppression are the known predisposing factors for mucormycosis (*1*). During the second wave of the COVID-19 pandemic (April–June 2021), a large number of cases of COVID-19–associated mucormycosis (CAM) were reported globally, primarily in India (*2*–*5*). The explanation for this outbreak of CAM in India remains unclear. Diabetes mellitus and glucocorticoids (used for treating COVID-19) have been identified as risk factors for CAM (*2*,*6*). Other factors proposed in the pathogenesis of CAM include altered iron metabolism, the severity of COVID-19, and immune dysfunction resulting from COVID-19 (e.g., lymphopenia and others) (*7*,*8*).

A high burden of Mucorales (in the hospital and outdoor environments) has been reported in India during and even before the CAM epidemic (*9*,*10*). We also found no difference in the Mucorales species causing mucormycosis before and during the COVID-19 pandemic (*2*,*11*). The epidemiologic triad of agent, environmental, and host factors is helpful to explain the occurrence of a new illness or the recrudescence of an old disease (*6*,*8*,*9*). Because the data indicate no change in the environment or the agent (Mucorales), we hypothesized that COVID-19, its treatment, and specific host factors contributed to the CAM outbreak. 

We evaluated the risk factors and clinical outcomes of CAM in a nationwide study. The main objective of our study was to assess whether treatment practices in COVID-19 were associated with the occurrence of CAM. We also evaluate the factors associated with death from CAM at 12 weeks.

## Methods

### Study Design and Setting

We performed a multicenter (25 centers across India) case–control study during January 1–June 30, 2021 (Appendix Figure). We included CAM patients (cases) and at least 2 COVID-19 patients without mucormycosis (controls) for each case. Only those centers willing to provide data on >15 cases of mucormycosis during the outbreak were included (Appendix Table 1).

The Institute Ethics Committees approved the study protocol at the individual study sites. A consent waiver was granted because we used anonymized patient data for analysis. The study is reported according to the Strengthening the Reporting of Observational Studies in Epidemiology (STROBE) guidelines (Appendix) (*12*).

### Cases and Controls

We confirmed COVID-19 diagnosis by SARS-CoV-2 RNA positivity in respiratory specimens by reverse transcription PCR or a positive rapid antigen test. We included only confirmed (proven and probable) cases of mucormycosis diagnosed and managed at the individual centers (*13*). Participating centers were tertiary care referral hospitals or research institutes equipped with the facilities required to evaluate and manage mucormycosis. We evaluated patient samples at the study sites by conventional microscopy (KOH-calcofluor method), culture, histopathology, or molecular diagnostic techniques, as appropriate. We identified positive cultures on the basis of macroscopic and microscopic characteristics of the growth or by sequencing of the internal transcribed spacer region of rDNA. We subjected tissue samples for histopathological examination and used hematoxylin and eosin, periodic acid Schiff, or Gomori's methenamine silver stain. We diagnosed invasive mucormycosis on the basis of existing guidelines and as previously described (*11*,*13*). In brief, we categorized participants’ illness as proven mucormycosis if Mucorales were isolated or broad ribbon-like aseptate hyphae were demonstrated from sterile sites or deep tissue biopsy. We defined probable mucormycosis in the presence of host risk factors, consistent imaging (e.g., reversed halo sign, thick-walled cavity, and others on computed tomography), and the demonstration of Mucorales by either microscopy or culture from a nonsterile site (sputum, nasal smear, or bronchoalveolar lavage fluid) (*13*,*14*). We defined disseminated mucormycosis as >1 noncontiguous site being involved. We excluded cases without microbiological or pathological confirmation of mucormycosis. We considered isolation of Mucorales from respiratory secretions without compatible host factors, clinical features, or radiology as colonization, and these cases were not included. We further classified CAM as concurrent (occurrence of mucormycosis within 7 days before or after diagnosing COVID-19) or nonconcurrent (after 7 days but within 3 months of COVID-19 diagnosis).

We enrolled >2 age-matched (+5 years) persons with confirmed COVID-19 as controls for each CAM case. The controls were selected randomly by the individual centers. We also reviewed patient records or contacted control-patients by telephone 3 months after COVID-19 diagnosis to ensure CAM had not developed.

Patients were managed per the local institutional protocol and the treating physician’s discretion. Mucormycosis was treated according to the standard recommendations, subject to the availability of drugs and other factors (*1*,*15*). We recorded the first antifungal medication (amphotericin B, posaconazole, isavuconazole, or a combination) used for managing mucormycosis and whether lack of availability of the intended drug led to the use of an alternate agent. We defined the use of >2 antifungal agents effective against Mucorales within the first 14 days of CAM as primary combination antifungal therapy. We also noted whether >1 doses were missed because of drug unavailability.

### Exposures and Confounding Variables

The primary exposure variable was the type of treatment offered for COVID-19. The therapies consisted of glucocorticoids, remdesivir, tocilizumab, baricitinib, zinc supplements, antibacterial agents, and antifungal therapy (before onset of mucormycosis). We defined inappropriate glucocorticoid therapy as use of systemic glucocorticoids for COVID-19 without related hypoxemia. We recorded information on the dose and duration of glucocorticoids used for COVID-19 and calculated the cumulative dose of glucocorticoids by multiplying the number of days of therapy and the dose used (dexamethasone equivalent).

We accounted for potential confounding variables pertaining to CAM by retrieving demographic and clinical information about COVID-19 illness from patient records. That information was sex; place of residence (rural or urban); investigations performed during admission (the first available values) for acute COVID-19 illness, including glycated hemoglobin (HbA1C), plasma glucose, complete blood count, serum ferritin, C-reactive protein, and neutrophil-to-lymphocyte ratio; host factors, including diabetes mellitus (labeled as recent-onset if diagnosed during the current admission and there was no previous history of diabetes), diabetic ketoacidosis during admission for COVID-19, hematological malignancy, stem cell or organ transplantation, and immunosuppressive therapy (for indications unrelated to COVID-19); chronic comorbidities (chronic liver, kidney, lung, and other diseases); hypoxemia during acute COVID-19 (<94% oxygen saturation while breathing ambient air or requiring supplemental oxygen); and the need for mechanical ventilation.

We also evaluated factors associated with 12-week mortality in CAM patients. We collected the following data: time to diagnosis of mucormycosis after confirmed COVID-19, diagnosis of mucormycosis (microscopy, culture, histopathology), anatomic site of involvement (rhino-orbital mucormycosis, pulmonary, or others), treatment details (antifungal therapy, surgery, and others), and outcome at 6 and 12 weeks.

### Study Size

We assumed the primary exposure of interest (treatment practices, primarily glucocorticoids) would be present in 60% of controls and 70% of cases. The estimated sample size was 374 cases and 747 controls for a case: control enrollment ratio of 1:2, at a power of 90% for detecting 1.25 odds (in cases than controls). We planned a convenient sample size by enrolling >15 consecutive CAM cases from each participating center.

### Statistical Analysis

We analyzed data by using the commercial statistical package SPSS Statistics 22.0 (IBM, https://www.ibm.com). As appropriate, descriptive data are presented as frequencies, means and SDs, or medians and interquartile ranges. We compared categorical variables by using the χ^2^ test or Fisher exact test and analyzed the differences between continuous data by using the Student *t*-test or Mann-Whitney U test, as appropriate. We considered a p value <0.05 significant.

We imputed the missing data for performing the subsequent logistic regression analysis. The pattern of missing data (missing at random or not) was ascertained. We then performed multiple imputations (50 iterations) by using the Markov Chain Monte Carlo method (Appendix Table 2). We performed multivariate logistic regression analyses to identify the factors associated with the development of CAM (vs. COVID-19 controls) and ascertain factors associated with 12-week mortality in persons with CAM. The variables included in the logistic regression model were decided on the basis of the univariate analysis and biologic plausibility. The strength of association was reported as an adjusted odds ratio (aOR) with 95% CI. To elucidate the confounding resulting from the difference in severity among the controls and cases, we repeated the multivariate analysis in the following subgroups: nonhypoxemic versus hypoxemic COVID-19 control-patients and COVID-19 control-patients with and without comorbidities. We also report the sensitivity analysis on the available data (complete case analysis) for the logistic regression.

## Results

We included 1,733 CAM case-patients and 3,911 control-patients in the study (Table 1). Of the study participants, 684 did not require hospitalization for the management of acute COVID-19. 

**Table 1:** Baseline features of CAM case-patients and COVID-19 control-patients at admission for COVID-19, India, January–June 2021*

Category	Controls, n = 3,911	CAM, n = 1,733	p value
Age, y, mean (SD)	52.7 (13.5)	52.6 (12.5)	0.66
Male sex	2,738/3,911 (70.0)	1,285/1,733 (74.1)	0.002
Rural residence, n = 3,924	309/2,615 (11.8)	360/1,309 (27.5)	0.0001
Risk factors for mucormycosis			0.0001
None	2,076/3,911 (53.1)	316/1,733 (18.2)	
1 risk factor	1,743/3,911 (44.5)	1,374/1,733 (79.3)	
>1 risk factors	92/3,911 (2.4)	43/1,733 (2.5)	
Details of potential risk factors for mucormycosis†			
Diabetes mellitus	1,763/3,911 (45.1)	1,402/1,733 (80.9)	0.0001
Hyperglycemia at admission, n = 5,236	998/3,625 (27.5)	758/1,611 (47.1)	0.0001
Plasma glucose at admission, mg/dL, mean (SD), n = 3,487	195 (94)	235 (106)	0.0001
Glycated hemoglobin, mean (SD), n = 1,856	7.7 (2.5)	10.1 (2.9)	0.0001
Duration of diabetes, y, mean (SD), n = 861	9.6 (9.5)	8.4 (6.8)	0.04
Recent onset of diabetes mellitus	319/1,763 (18.1)	246/1,402 (17.5)	0.66
DKA at the time of admission for COVID-19	48/1,763 (2.7)	73/1,402 (5.2)	0.0003
Renal transplant	36/3,911 (0.9)	31/1,733 (1.8)	0.005
Bone marrow transplant	0/3,911 (0)	1/1,733 (0.1)	0.31
Hematological malignancy	36/3,911 (0.9)	6/1,733 (0.3)	0.02
Immunosuppressive therapy	76/3,911 (1.9)	22/1,733 (1.3)	0.07
HIV	7/3,911 (0.2)	6/1,733 (0.3)	0.23
Others‡	2/3,911 (0.0)	2/1,733 (0.1)	0.23
Comorbidities			
Any comorbidity	828/3,911 (21.5)	265/1,733 (15.3)	0.0001
Coronary artery disease	285/3,911 (7.3)	126/1,733 (7.3)	0.98
Chronic kidney disease	284/3,911 (7.3)	98/1,733 (5.7)	0.03
Chronic heart failure	59/3,911 (1.5)	17/1,733 (1.0)	0.11
Chronic liver disease	71/3,911 (1.8)	13/1,733 (0.8)	0.002
Chronic respiratory disease	104/3,911 (2.7)	17/1,733 (1.0)	0.0001
Others§	151/3,911 (3.9)	35/1,733 (2.0)	0.0001
Laboratory parameters during COVID-19 illness			
Hemoglobin, g/dL, mean (SD), n = 4,506	12.2 (2.4)	12.1 (2.2)	0.11
Total leukocyte count, cells/µL, mean (SD), n = 4,501	9,853 (6,844)	11,396 (6,110)	0.0001
Median absolute lymphocyte count, cells/µL (IQR), n = 4,129	1,135 (720–1,706)	1,275 (803–1,833)	0.0001
Median absolute neutrophil count, cells/µL (IQR), n = 4,071	6,177 (3,658–10,244)	7,858 (4,943–11,867)	0.0001
Median NLR (IQR), n = 4,061	5.5 (2.7–11.4)	5.7 (3.2–11.7)	0.04
Platelet count, × 10^3^/µL, mean (SD), n = 4,454	222 (107)	240 (105)	0.0001
Median C-reactive protein mg/dL (IQR), n = 3,972	26.7 (8.4–79.3)	48.8 (20–102.5)	0.0001
Median serum ferritin, µg/L (IQR) n = 3,168	454 (189–977)	580 (238–1,052)	0.02
Details of COVID-19 illness			
Hypoxemia, n = 5,476	2,100/3,851 (54.5)	751/1,625(46.2)	0.0001
ICU admission, n = 5,425	1,551/3,809 (40.7)	331/1,616 (20.5)	0.0001
Mechanical ventilation, n = 5,376	1,126/3,765 (29.9)	153/1,611 (9.5)	0.0001
Management during COVID-19			
Glucocorticoid therapy, n = 5,431	2,690/3,827 (70.3)	1,200/1,604 (74.8)	0.001
Glucocorticoid use in the absence of hypoxemia, n = 5,021	789/3,532 (22.3)	509/1,489 (34.2)	0.0001
Median cumulative dose of glucocorticoids in milligram equivalent of dexamethasone (IQR), n = 2,809	52.8 (30–84)	62.6 (30.2–120)	0.0001
Median no. days on glucocorticoid treatment (IQR), n = 2,887	8 (5–12)	10 (6.3–14)	0.0001
Zinc supplementation, n = 5,179	1,502/3,633 (41.3)	741/1,546 (47.9)	0.0001
Remdesivir, n = 5,167	1,785/3,631 (49.2)	317/1,536 (20.6)	0.0001
Tocilizumab, n = 5,167	72/3,631 (2.0)	37/1,536 (2.4)	0.41
Baricitinib, n = 5,167	38/3,631 (1.0)	13/1,536 (0.8)	0.50
Antibacterial therapy, n = 5,396	2,467/3,841 (64.2)	952/1,555 (61.2)	0.04
Antifungal therapy before CAM, n = 5,039	174/3,513 (5.0)	68/1,526 (4.5)	0.45

*Values are no. observed/total no. (%) unless otherwise indicated. CAM, COVID-19–associated mucormycosis; DKA, diabetic ketoacidosis; DM, diabetes mellitus; ICU, intensive care unit; IQR, interquartile range; NLR, neutrophil-to-lymphocyte ratio. †A single person might have had >1 risk factor; hence the numbers do not sum to 5,644. ‡Others include neutropenia (n = 3) and primary immunodeficiency (n = 1). §Others include neurologic, endocrinologic, and rheumatologic illnesses.

### Exposure

Glucocorticoids were used for COVID-19 in 71.6% (3,890/5,431) patients (cumulative dose range 0.76–679.53 mg dexamethasone equivalent), and their use was more frequent in CAM case-patients. The median dose and duration of glucocorticoid use was also higher in case-patients than control-patients (Table 1). The proportion of persons receiving glucocorticoids in the absence of hypoxemia (inappropriate use) was even higher in CAM patients (34.2% vs. 22.3%; p = 0.0001) than control-patients. In addition, the percentage of persons receiving more than the recommended dose (>60 mg of dexamethasone or equivalent) of glucocorticoids was higher among CAM case-patients than COVID-19 control-patients (Figure), both for hypoxemic (269/439 [61.3%] vs. 683/1389 [49.2%]; p = 0.0001) and nonhypoxemic (124/329 [37.7%] vs. 143/633 [22.6%]; p = 0.0001) persons. Zinc supplementation during COVID-19 illness was higher among case-patients than controls (47.9% vs. 41.3%; p = 0.0001). Remdesivir (49.2% vs. 20.6%; p = 0.0001) and antibacterial therapy (64.2% vs. 61.1%; p = 0.03) were more commonly used in controls. No difference was noted in the proportion of participants receiving tocilizumab, baricitinib, or antifungal therapy before the development of CAM.

**Figure:**
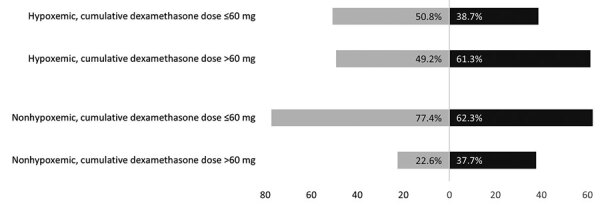
Percentages of hypoxemic and nonhypoxemic participants receiving cumulative glucocorticoid doses above and below the current recommendations (cumulative dexamethasone dose equivalent to 60 mg) as determined in multicenter case-control study of COVID-19–associated mucormycosis outbreak, India, January–June 2021. Case-patients are shown in black and control-patients are shown in gray. More case-patients than control-patients had received higher-than-recommended doses of glucocorticoids.

### Confounders

Chronic comorbidities (chronic liver, kidney, lung, and other diseases) were present in 37% of study participants and were seen more often in controls than in CAM patients. Significantly higher plasma glucose, neutrophil-to-lymphocyte ratio, platelet count, C-reactive protein, and serum ferritin were seen in CAM patients during their hospitalizations for COVID-19. We identified >1 host factors for mucormycosis in 46.9% of controls versus 81.8% of case-patients (p = 0.0001). The proportion of diabetes mellitus was significantly higher in case-patients (80.9% vs. 45.1%; p = 0.0001) than in controls. Recent-onset diabetes mellitus was similar in both the study groups, whereas diabetic ketoacidosis was significantly more common among CAM than in the controls (5.2% vs. 2.7%; p = 0.0003). We found hypoxemia during acute COVID-19 (54.5% vs. 46.2%), admission to an intensive care unit (40.7% vs. 20.5%), and mechanical ventilation (29.9% vs. 9.5%) significantly higher among control-patients.

### Association of COVID-19 Treatment Practices with CAM

We found the cumulative dose of glucocorticoid used (OR 1.006, 95% CI 1.004–1.007) and zinc supplementation (OR 2.76, 95% CI 2.24–3.40) independently associated with CAM, in addition to elevated C-reactive protein, host factors (renal transplantation, diabetes mellitus, diabetic ketoacidosis during COVID-19), and rural residence (Table 2). Hypoxemia during COVID-19 and comorbidities were associated with lower odds of CAM. The results were similar on a complete case analysis (Appendix Table 3). Because the proportion of persons with hypoxemia and comorbidities was higher in COVID-19 controls than in CAM patients, we also performed a subgroup analysis (hypoxemic vs. nonhypoxemic controls and any comorbidity vs. no comorbidity; Appendix Tables 4, 5). We found the primary exposure of interest (COVID-19 treatment factors) significantly associated with CAM in both the subgroups (Appendix Tables 4, 5).

**Table 2:** Multivariate logistic regression analysis showing factors associated with CAM, India, January–June 2021*

Parameter	Adjusted OR (95% CI)	p value
Female sex	0.92 (0.74–1.14)	0.46
Rural residence	2.88 (2.12–3.79)	0.0001
Risk factor		
No risk factor	Referent	
Diabetes mellitus	6.72 (5.45–8.28)	0.0001
Renal transplantation	7.58 (3.31–17.40)	0.0001
Others†	1.20 (0.67–2.18)	0.54
Presence of any comorbidity	0.50 (0.39–0.63)	0.0001
Hypoxia during COVID-19	0.26 (0.21–0.32)	0.0001
Diabetic ketoacidosis during COVID-19	4.41 (2.03–9.60)	0.0001
Cumulative glucocorticoid dose for COVID-19‡	1.006 (1.004–1.007)	0.0001
Zinc supplementation during COVID-19	2.76 (2.24–3.40)	0.0001
C-reactive protein at admission	1.004 (1.002–1.006)	0.0001
Serum ferritin, µg/L	1.00 (1.00–1.00)	0.21
Neutrophil-to-lymphocyte ratio	1.0 (0.99–1.01)	0.92

*CAM, COVID-19–associated mucormycosis. †Includes malignancies, hematological malignancies, immunosuppressive therapy, HIV, and others. ‡In milligram equivalent of dexamethasone.

We also assessed the association of inappropriate glucocorticoid therapy (i.e., glucocorticoids administered in the absence of hypoxemia) with CAM. In an alternative logistic regression model, we replaced glucocorticoid doses with inappropriate glucocorticoid therapy (Appendix Table 6) and found inappropriate therapy also significantly associated with CAM.

### Additional Analyses of Clinical Features, Diagnosis, Treatment, and Outcome of CAM

CAM occurred nonconcurrently (>7 days after COVID-19 diagnosis) in 1,405 (81.7%) of 1,720 persons; the remaining 315 (18.3%) cases were concurrent with the COVID-19 illness (within 7 days of diagnosis). The duration between COVID-19 and the diagnosis of CAM was shorter for those hospitalized for COVID-19 (mean 20, 95% CI 19–21 days) versus those isolated at home (mean 25, 95% CI 24–27 days). The median duration between symptoms of CAM and confirmation of the diagnosis was 6 (interquartile range 3–10) days (n = 1,024 persons). Rhino-orbital mucormycosis accounted for 92.4% of the cases, followed by pulmonary (7%) and other sites (0.6%). The proportion of pulmonary mucormycosis was higher in persons who had undergone organ transplants (17.2%) and persons with hematological malignancies (33.3%) than in those with diabetes mellitus (6.4%). Of the 1,602 patients with rhino-orbital mucormycosis, we noted intracranial extension in 261 (16.3%) cases. Nearly two thirds (1,143/1,733; 66.0%) of the CAM patients had evidence of mucormycosis from >1 sample (smear, culture, or histopathology) (Table 3). The most common etiologic agent causing mucormycosis was *Rhizopus* spp*.* (mainly *R. arrhizus*)*.* The other reported organisms included *Mucor* and *Rhizomucor* spp., and rarely *Cunninghamella*, *Syncephalastrum*, *Apophysomyces*, *Lichtheimia* spp., and others.

**Table 3:** Diagnosis, treatment practices, and survival in patients with CAM, India, January–June 2021*

Parameter	No. observed/total no. (%)
Site of mucormycosis†	
Rhino-orbito-cerebral	
Sinus	1,526/1,733 (88.1)
Orbit	789/1,733 (45.5)
Palate	373/1,733 (21.5)
Jaw	315/1,733 (18.2)
Brain	261/1,733 (15.1)
Blackening of skin over face	102/1,733 (5.9)
Cavernous sinus	44/1,733 (2.5)
Skull base	65/1,733 (3.8)
Pulmonary†	122/1,733 (7.0)
Cutaneous or soft tissue	5/1,733 (0.3)
Gastrointestinal	2/1,733 (0.1)
Renal†	2/1,733 (0.1)
Diagnosis of mucormycosis	
Microscopy alone	352/1,733 (20.3)
Culture growth of Mucorales alone	61/1,733 (3.5)
Histopathology alone	177/1,733 (10.2)
>1 evidence (smear, culture, or histopathology) of mucormycosis	1,143/1,733 (66.0)
Treatment practices	
Intended therapy could not be administered	321/1,526 (21.0)
Missed doses due to drug non-availability	248/1,457 (17.0)
Primary therapy	
Any formulation of amphotericin B‡	1,634/1,733 (94.3)
Primary combination therapy§	
Any combination	699/1,733 (41.6)
Amphotericin B and posaconazole	541/699 (77.4)
Amphotericin B and isavuconazole	121/699 (17.3)
Amphotericin B and isavuconazole or posaconazole	37/699 (5.3)
Surgery	
Combined medical and surgical treatment	1,449/1,773 (83.6)
Survival	
Death by 6 weeks	442/1,546 (28.6)
Death by 12 weeks	473/1,471 (32.2)

*CAM, COVID-19–associated mucormycosis †Total number does not sum to 1,733 since patients might have had involvement at >1 site. There were 18 cases of disseminated mucormycosis (17 had pulmonary in addition to rhino-orbital, while 1 person had rhino-orbito-cerebral and renal mucormycosis). ‡Of the 1,634 persons receiving amphotericin B, liposomal amphotericin B alone was used in 1,210 (74.1%) patients, amphotericin B deoxycholate in 143 (8.7%) patients, amphotericin B lipid emulsion in 21 (1.3%) patients, >1 formulation in 236 (14.4%) patients, and the information was not clear in 24 (1.5%) patients. §Primary therapy with a combination of amphotericin and isavuconazole or posaconazole within the first 14 days was used in 699/1,733 (41.6%) patients.

The treatment of CAM varied widely and was influenced by antifungal drug availability. The intended antifungal agent could not be administered in 21.0% (321/1,526) of cases, and >1 dose was missed because the antifungal drugs were unavailable in 17% (248/1,547) of CAM cases. Amphotericin B was the most prescribed antifungal agent. A combination of antifungal agents was used for primary therapy in 41.6% of patients. Surgery was performed in most case-patients (1,449/1,733; 83.6%). The mortality rate at 6 weeks (data available for 89.2% of cases) was 28.6% and at 12 weeks (data available for 84.9% cases) was 32.2%.

We found surgical resection and primary antifungal combination therapy independently associated with better odds of survival at 12 weeks (Table 4). We also found increasing age and intracranial extension associated with worse odds of survival after adjusting for sex, comorbidities, and COVID-19–related hypoxemia. The results were similar on a complete case analysis (Appendix Table 7).

**Table 4:** Factors associated with death at 12 weeks in persons with CAM, India, January–June 2021*

Parameter	Adjusted odds ratio (95% CI)	p value
Age	1.02 (1.01–1.04)	0.0001
Sex	1.00 (0.74–1.34)	0.99
Risk factor		
No risk factor	Referent	
Diabetes mellitus	1.27 (0.93–1.74)	0.13
Renal transplantation	2.66 (1.04–6.81)	0.04
Others†	1.51 (0.55–4.18)	0.42
Presence of any comorbidity	1.38 (0.97–1.97)	0.08
Hypoxemia during COVID-19 illness	1.31 (0.93–1.83)	0.12
Site of mucormycosis		
Rhino-orbital mucormycosis	Referent	
Rhino-orbital mucormycosis with brain involvement	2.30 (1.66–3.19)	0.0001
Other sites‡	1.44 (0.90–2.32)	0.13
Primary combination medical therapy	0.53 (0.37–0.77)	0.001
Combined medical and surgical treatment	0.20 (0.14–0.27)	0.0001

*CAM, COVID-19–associated mucormycosis. †Includes hematological malignancies, immunosuppressive therapy, and HIV infection. ‡Includes pulmonary, gastrointestinal, disseminated, and renal mucormycosis.

## Discussion

In this large case-control study, we found that glucocorticoid use and zinc supplementation in the treatment of COVID-19 were significantly associated with CAM. In addition, several host factors for mucormycosis (i.e., renal transplantation, diabetes mellitus, and diabetic ketoacidosis), elevated C-reactive protein, and rural residence were also associated with CAM. The unprecedented rise in the number of CAM cases during the second wave of the COVID-19 pandemic indicates that COVID-19 or its treatment had a role in causing CAM (*2*,*16*).

We provide strong evidence to incriminate glucocorticoid therapy in CAM even after adjusting for host factors. Our results strengthen the current recommendation of avoiding glucocorticoid use in COVID-19 patients not experiencing hypoxemia (*17*,*18*). More critically, we found that the cumulative glucocorticoid dose is also a contributory factor for CAM. Thus, even in hypoxemic COVID-19 patients, glucocorticoids should be used judiciously (dexamethasone at a dose of 6 mg 1×/d for up to 10 days or until hospital discharge, whichever is earlier) (*19*). We also found zinc supplementation an independent factor associated with CAM. A small study suggested a protective role of zinc in CAM (*6*), but 2 other studies found an association between zinc and CAM (*20*,*21*). The biologic plausibility (*22*) and in vitro evidence of abundant growth of Mucorales strains (isolated from CAM patients) demonstrated on zinc-supplemented media supports the possible role of zinc in causing CAM (*20*). Although we found a few factors related to the severity and treatment of COVID-19 in the development of CAM, we did not evaluate the role of COVID-19–related immune dysfunction in this study. Nonetheless, we provide weak indirect evidence implicating the severity of COVID-19 illness (elevated C-reactive protein) in the development of CAM. Finally, rural residence was significantly associated with CAM and might be attributed to higher levels of fungal spores in the rural environment (*23*–*26*). 

The time to develop CAM was significantly shorter in hospitalized persons than in persons isolated at home. This finding could suggest hospital-acquired mucormycosis; however, the hospitalization could simply mean that severe COVID-19 led to mucormycosis early in the course of illness or, more likely, that CAM was diagnosed earlier simply because these persons were hospitalized. Based on a few media reports, 1 review hypothesized that the burning of cow dung led to the mucormycosis outbreak in India (*27*). However, a recent experimental aeromycological study found no evidence for this theory (*25*).

Several case-control studies (n = 13) have assessed the risk factors associated with CAM (Appendix Table 8) (*6*,*7*,*20*,*21*,*28*–*36*). In 8 studies (sample sizes ranging from 46 to 870), risk factors for CAM were assessed using multivariate analysis (*6*,*7*,*29*–*33*,*35*). Diabetes mellitus and glucocorticoid therapy were shown to be the major contributors for CAM in the case–control studies and large case series (*2*,*3*,*6*,*21*,*35*,*37*). Further, in addition to zinc and elevated inflammatory markers (C-reactive protein), which were also associated with CAM in this study, the use of cloth masks, nasal washing during COVID-19, and elevated serum glucose-regulated protein 78 were other possible associations described in smaller case–control studies (*29*,*30*,*33*). In our study, mean glycated hemoglobin values were significantly higher in the CAM cases. In another study, optimal glycemic control and adherence to low-dose glucocorticoid protocol eliminated the occurrence of mucormycosis in a COVID-19 intensive care unit even during the surge in CAM cases (*38*). Unfortunately, because of the overwhelming case burden, many COVID-19 patients were taking various prescription or over-the-counter medications (including glucocorticoids and zinc) unmonitored, which probably contributed to the outbreak.

We found lower rates of mortality in mucormycosis patients than in previous reports (*11*,*39*,*40*). The lower mortality rate in our study might be attributed to several factors. First, more severe forms of the disease, including pulmonary and disseminated mucormycosis, could have been underrepresented (*41*,*42*). For instance, pulmonary mucormycosis accounted for only 7% of the cases in this study (vs. 13% in a pre-COVID-19 large multicenter study from India) (*11*). Second, the increased awareness about mucormycosis because of the outbreak led to timely diagnosis (median time to diagnosis 6 days) and surgical intervention in a higher percentage of cases (84%) than in the pre–COVID-19 era (62%) (*11*). Also, the predisposing factors in CAM cases, such as glucocorticoid therapy and hyperglycemia, were mostly reversible. The use of primary combination antifungal therapy might have contributed to improved outcomes. A previous multicenter observational study on CAM also indicated that combining amphotericin B with posaconazole might be associated with better outcomes than monotherapy (*2*). However, attributing the benefit of combination treatment to survival without a randomized clinical trial is difficult. Our results also confirm the existing knowledge that surgical treatment is associated with better outcomes in mucormycosis (*1*,*11*).

The first limitation of our study is that data were collected during the peak of the pandemic with limited resources, and some information was missing as a result. Although our study supports the definite association of glucocorticoids with CAM, the same might not be accurate for zinc. Zinc might be synergistic to glucocorticoids or other factors in the development of CAM. However, it was an independent risk factor across different subgroups and in different multivariate models. Although we could obtain information on zinc supplementation during the treatment of COVID-19, the wide variation in prescription practice, over-the-counter availability of drugs, and use of different formulations and dosages precluded establishing a dose-response relationship between zinc and CAM. Thus, prospective studies and animal experiments are warranted to establish the association of zinc with CAM. Even though we enrolled a large number of control-patients, the control-patients were sicker than case-patients. This difference in severity of COVID-19 symptoms influenced a few of our results. For instance, the presence of any comorbidity and the need for mechanical ventilation were associated with a lower risk for CAM. One could argue that our association of the primary exposure variable with CAM is invalid because the control-patients critically ill with COVID-19 might not have survived long enough for CAM to develop. Imperfect matching for COVID-19 severity is thus a major limitation. To adjust for the severity of COVID-19, we performed a subgroup analysis in which we compared the CAM patients with either hypoxemic or nonhypoxemic COVID-19 control-patients. We found the COVID-19 treatment factors (primary exposure) remained significantly associated with CAM in both groups (Appendix Table 4). We restricted our data collection to information that could be accessed reliably despite the pandemic. Thus, we cannot exclude residual confounders. Not all the participating centers could provide the desired number of control subjects, and there was variation in mortality reported from different centers (Appendix Table 1). A referral bias to the participating study centers and underrecognition of certain forms of mucormycosis (pulmonary and others) could have influenced our observations. We could not assess the burden of CAM among COVID-19 case-patients. Also, we included cases diagnosed by conventional microbiological techniques, and we might have missed several presumed cases of mucormycosis. However, the objective of our study was to evaluate the risk factors in a case-control model, and hence we included confirmed cases only. Finally, the results might not be generalizable because the information is from just 1 country. The key strength of our study is the large sample size and representation from across the country, which lends credibility to our observations.

In conclusion, we found several treatment practices associated with CAM in addition to rural residence and host factors. Our results suggest the judicious use of COVID-19 therapies and optimal glycemic control to prevent CAM.

Appendix Additional information about multicenter case-control study of COVID-19–associated mucormycosis outbreak, India
