# Supplementary material for: Multicenter Case–Control Study of COVID-19–Associated Mucormycosis Outbreak, India
Source: Emerg Infect Dis. 2023 Jan;29(1):8–19. doi: 10.3201/eid2901.220926 (PMC9796192; doi:10.3201/eid2901.220926)
Supplement: Appendix — Additional information about multicenter case-control study of COVID-19–associated mucormycosis outbreak, India [file 22-0926-Techapp-s1.pdf]

Article DOI: https://doi.org/10.3201/eid2901.220926

Multicenter Case-Control Study of COVID-19–Associated Mucormycosis Outbreak, India

Appendix

Study Sites and Data Collection

The study was conducted after the Institute ethics committee of each study site approved the project. We circulated a standardized case-record form to the participating centers, and data was extracted manually from the individual patient case records by research personnel. We gathered anonymized patient data and ensured confidentiality. We assigned unique identification numbers for each form and entered them into a freely available mobile-based data gathering platform (Epicollect5). Data entry was done by trained clinical research staff at the coordinating center (Postgraduate Institute of Medical Education and Research [PGIMER], Chandigarh) under the supervision of four investigators (V.M., R.A., S.M.R., A.C.). Double entry of all the CAM cases was performed by two investigators (V.M. and R.A.). We imported the data to a spreadsheet before transferring it to statistical software for data analysis.

References

1. Muthu V, Kumar M, Paul RA, Zohmangaihi D, Choudhary H, Rudramurthy SM, et al. Is there an association between zinc and COVID-19–associated mucormycosis? Results of an experimental and clinical study. Mycoses. 2021;64:1291–7. PubMed https://doi.org/10.1111/myc.13365

2. Bansal SB, Rana A, Babras M, Yadav D, Jha P, Jain M, et al. Risk factors and outcomes of COVID associated mucormycosis in kidney transplant recipients. Transpl Infect Dis. 2022;24:e13777. PubMed https://doi.org/10.1111/tid.13777

3. Arora U, Priyadarshi M, Katiyar V, Soneja M, Garg P, Gupta I, et al. Risk factors for coronavirus disease–associated mucormycosis. J Infect. 2022;84:383–90. PubMed https://doi.org/10.1016/j.jinf.2021.12.039

4. Karat S, Lobo AC, Satish D, Devaraj R, Manjooran RR, Nithyanandam S. Uncontrolled diabetes mellitus exacerbated by COVID-19–induced inflammation is the risk factor for COVID-19–associated rhino-orbito-cerebral mucormycosis: a matched pair case-control study. Indian J Ophthalmol. 2022;70:3096–101. PubMed https://doi.org/10.4103/ijo.IJO_448_22

5. Kumar HM, Sharma P, Rudramurthy SM, Sehgal IS, Prasad KT, Pannu AK, et al. Serum iron indices in COVID-19-associated mucormycosis: a case-control study. Mycoses. 2022;65:120–7. PubMed https://doi.org/10.1111/myc.13391

6. Kumar S, Acharya S, Jain S, Shukla S, Talwar D, Shah D, et al. Role of zinc and clinicopathological factors for COVID-19–associated mucormycosis (CAM) in a rural hospital of central India: a case-control study. Cureus. 2022;14:e22528. PubMed https://doi.org/10.7759/cureus.22528

7. Muthu V, Dhaliwal M, Sharma A, Nair D, Kumar HM, Rudramurthy SM, et al. Serum glucose-regulated protein 78 (GRP78) levels in COVID-19-associated mucormycosis: results of a case-control study. Mycopathologia. 2022;187:355–62. PubMed https://doi.org/10.1007/s11046-022-00645-6

8. Pandit AK, Tangri P, Misra S, Srivastava MVP, Bhatnagar S, Thakar A, et al. Mucormycosis in COVID-19 patients: a case-control study. Microorganisms. 2022;10:1209. PubMed https://doi.org/10.3390/microorganisms10061209

9. Patel AK, Bakshi H, Shah K, Patel S, Patel T, Patel K, et al. Risk factors for COVID-19 associated mucormycosis in India: a case control study. Med Mycol. 2022;60:myac044. PubMed https://doi.org/10.1093/mmy/myac044

10. Ponnaiah M, Ganesan S, Bhatnagar T, Thulasingam M, Majella MG, Karuppiah M, et al. Hyperglycemia and steroid use increase the risk of rhino-orbito-cerebral mucormycosis regardless of COVID-19 hospitalization: case-control study, India. PLoS One. 2022;17:e0272042. PubMed https://doi.org/10.1371/journal.pone.0272042

11. Popli H, Gupta A, Singh V, Agarwal V, Akilan R, Kumar A. Are low serum vitamin D levels a risk factor for advent of COVID-19 associated rhinocerebral mucormycosis: a preliminary case control study. Indian J Otolaryngol Head Neck Surg. 2022 Jan 11 [Epub ahead of print]. PubMed https://doi.org/10.1007/s12070-022-03080-7

12. Vasanthapuram VH, Gupta R, Adulkar N, Nair AG, Bradoo RA, Hegde R, et al. A fungal epidemic amidst a viral pandemic: risk factors for development of COVID-19 associated rhino-orbital-cerebral mucormycosis in India. Orbit. 2022 Feb 22 [Epub ahead of print]. PubMed https://doi.org/10.1080/01676830.2021.2020851

13. Yesupatham ST, Mohiyuddin SMA, Arokiyaswamy S, Brindha HS, Anirudh PB. Estimation of Ferritin and D-Dimer levels in COVID-19 patients with mucormycosis: a cross-sectional study. J Clin Diagn Res. 2022;16:BC12–5. https://doi.org/10.7860/JCDR/2022/52844.15908

Appendix Table 1. Details of the participating center and the number (percentage) of COVID-19 controls and COVID-19-associated mucormycosis (CAM) cases

Center no.	Participating center	COVID-19 controls	CAM	Mortality in CAM, n/N (%)
1	Postgraduate Institute of Medical Education and Research, Chandigarh	1084 (27.7)	327 (18.9)	114/250 (45.6)
2	Kovai Medical Center, Coimbatore	425 (10.9)	86 (5.0)	20/73 (27.4)
3	Avron Hospitals, Ahmedabad	229 (5.9)	169 (9.8)	27/169 (16)
4	Sterling hospital, Ahmedabad	293 (7.5)	100 (5.8)	23/100 (23)
5	Sir Gangaram Hospital, New Delhi	254 (6.5)	123 (7.1)	28/123 (22.8)
6	Kingsway Hospital, Nagpur	185 (4.7)	97 (5.6)	12/92 (13)
7	Sawai Man Singh Medical College, Jaipur	100 (2.6)	119 (6.9)	15/49 (30.6)
8	Asian Institute of gastroenterology, Hyderabad	177 (4.5)	36 (2.1)	3/36 (8.3)
9	St. John's National Academy of Health Sciences, Bengaluru	146 (3.7)	64 (3.7)	30/45 (66.7)
10	Care Institute of Medical Sciences, Ahmedabad, Gujarat	132 (3.4)	66 (3.8)	11/66 (16.7)
11	All India Institute of Medical Sciences, Bhubaneshwar	124 (3.2)	42 (2.4)	10/42 (23.8)
12	Yashoda Hospital, Hyderabad	102 (2.6)	61 (3.5)	17/60 (28.3)
13	Royal Care Hospital, Coimbatore	113 (2.9)	30 (1.7)	13/29 (44.8)
14	Apollo Hospitals, Chennai	105 (2.7)	37 (2.1)	15/37 (40.5)
15	Government Medical College, Patiala	89 (2.3)	46 (2.7)	24/46 (52.2)
16	Poona Hospital Research center, Pune	71 (1.8)	32 (1.8)	8/32 (25)
17	Apollo Hospitals, Bengaluru	86 (2.2)	85 (4.9)	22/85 (25.9)
18	Kokilaben Hospital, Mumbai	50 (1.3)	25 (1.4)	3/25 (12)
19	Research and Referral (Army) hospital, Delhi	44 (1.1)	28 (1.6)	7/28 (25)
20	Government Medical College, Chandigarh	18 (0.5)	52 (3.0)	26/52 (50)
21	Global Hospital, Mumbai	33 (0.8)	33 (1.9)	0/32 (0)
22	Deenanath Mangeshkar Hospital, Pune	20 (0.5)	29 (1.7)	3/29 (10.3)
23	City clinic and Bhailal Amin General Hospital, Vadodara	31 (0.8)	14 (0.8)	7/14 (50)
24	All India Institute of Medical Sciences, New Delhi	0	17 (1.0)	3/17 (17.6)
25	Institute of Infectious Disease & Critical Care, Surat	0	15 (0.9)	1/15 (6.7)
	Total	3,911	1,733	442/1,546 (28.6)

Appendix Table 2. Details of the multiple imputations performed using the Markov Chain Monte Carlo method

Parameter	Type	Missing Values	Imputed Values
Diabetic ketoacidosis at COVID-19 admission	Logistic Regression	108	5400
Mechanical ventilation for COVID-19	Logistic Regression	121	6050
Glucocorticoids for COVID-19	Logistic Regression	128	6400
Zinc supplementation for COVID-19	Logistic Regression	186	9300
Mortality at six weeks	Logistic Regression	186	9300
Appropriateness of glucocorticoids	Logistic Regression	243	12150
Mortality at twelve weeks	Logistic Regression	261	13050
Rural or urban	Logistic Regression	423	21150
Cumulative dose of glucocorticoids	Linear Regression	961	48050
Neutrophil-lymphocyte ratio	Linear Regression	1088	54400
C-reactive protein	Linear Regression	1175	58750
Serum ferritin	Linear Regression	1423	71150

Appendix Table 3. Multivariate logistic regression analysis showing factors associated with mucormycosis in COVID-19 (complete case analysis)

Parameter	Adjusted Odds ratio (95% CI)	p-value
Female sex	1.03 (0.65–1.63)	0.89
Rural residence	2.61 (1.61–4.21)	0.0001
Risk factor		
No risk factor	Reference category	
Diabetes mellitus	2.63 (1.72–4.05)	0.0001
Renal transplantation	8.20 (2.10–32.03)	0.002
Others*	1.01 (0.28–3.62)	0.98
Presence of any comorbid illness	0.67 (0.39–1.11)	0.14
Hypoxia during COVID-19	0.58 (0.37–0.92)	0.02
Diabetic ketoacidosis during COVID-19	6.37 (1.87–21.72)	0.003
Cumulative dose of glucocorticoids for COVID-19†	1.005 (1.003–1.007)	0.0001
Zinc supplementation during COVID-19	4.96 (3.21–7.65)	0.0001
C-reactive protein at admission	1.001 (0.99–1.004)	0.42
Serum ferritin, µg/L	1.00 (1.00–1.00)	0.73
Neutrophil-to-lymphocyte ratio	1.01 (0.99–1.03)	0.07

*Includes malignancies, hematological malignancies, immunosuppressive therapy, HIV, and others.
†In mg of dexamethasone equivalent

Appendix Table 4. Multivariate logistic regression analysis showing factors associated with mucormycosis in the subgroups of hypoxemic and non-hypoxemic COVID-19 controls

	Adjusted Odds ratio (95% CI)	
Parameter	Hypoxemic	Non-hypoxemic
Female sex	0.90 (0.72–1.12)	1.20 (0.86–1.68)
Rural residence	2.48 (1.88–3.27)*	3.17 (2.03–4.97)*
Risk factor		
No risk factor	Ref.	Ref.
Diabetes mellitus	6.13 (4.91–7.65)*	9.96 (7.16–13.86)*
Renal transplantation	8.74 (3.37–22.65)*	5.84 (1.76–19.33)†
Others‡	1.35 (0.73–2.48)	1.18 (0.53–2.64)
Presence of any comorbidity	0.55 (0.44–0.68)*	0.48 (0.35–0.65)*
Diabetic ketoacidosis during COVID-19	3.74 (1.64–5.35)*	4.43 (0.93–21.04)
Cumulative dose of glucocorticoids	1.00 (1.00–1.01)†	1.01 (1.00–1.01)*
Zinc supplementation during COVID-19	3.05 (2.42–3.85)*	1.80 (1.31–2.46)*
C-reactive protein at admission	1.00 (1.00–1.01)§	1.01 (1.00–1.01)†
Serum ferritin, µg/L	1.00 (1.00–1.00)	1.00 (1.00–1.00)
Neutrophil-to-lymphocyte ratio	0.98 (0.97–0.99)*	1.03 (1.01–1.05)†

*p value <0.0001.
†p value <0.001.
‡Includes malignancies, hematological malignancies, immunosuppressive therapy, HIV, and others.
§p value <0.05.

Appendix Table 5. Multivariate logistic regression analysis showing factors associated with mucormycosis in the subgroups of COVID-19 controls with and without comorbidities*

	Adjusted Odds ratio (95% CI)	
Parameter	With any comorbid illness	No comorbid illness
Female sex	0.95 (0.57–1.59)	0.91 (0.72–1.16)
Rural residence	1.89 (1.02–3.48)†	3.31 (2.42–4.51)‡
Risk factor		
No risk factor	Ref.	Ref.
Diabetes mellitus	7.34 (4.13–13.07)‡	6.52 (5.20–8.18)‡
Renal transplantation	10.0 (2.79–35.82)‡	6.02 (1.92–18.91)†
Others§	0.87 (0.25–3.01)	1.18 (0.53–2.64)
Hypoxemia during COVID-19	0.22 (0.13–0.36)‡	0.27 (0.21–0.34)‡
Diabetic ketoacidosis during COVID-19	3.04 (0.86–10.80)	5.65 (2.02–15.77)†
Cumulative dose of glucocorticoids	1.007 (1.003–1.011)‡	1.005 (1.003–1.007)‡
Zinc supplementation during COVID-19	3.94 (2.38–6.54)‡	2.55 (2.01–3.23)‡
C-reactive protein at admission	1.002 (0.99–1.01)	1.004 (1.002–1.007)‡
Serum ferritin, µg/L	1.00 (1.00–1.00)	1.00 (1.00–1.00)
Neutrophil-to-lymphocyte ratio	1.002 (0.99–1.02)	1.0 (0.99–1.01)

*Comorbid illnesses included chronic liver disease, chronic kidney disease, chronic heart failure, coronary artery disease, and chronic respiratory disease.
†p-value <0.05.
‡p value <0.0001.
§Includes malignancies, hematological malignancies, immunosuppressive therapy, HIV, and others.

Appendix Table 6. Multivariate logistic regression analysis showing factors associated with mucormycosis in COVID-19 (alternative model where glucocorticoid dose has been replaced by inappropriate glucocorticoid use [glucocorticoid use in those without hypoxemia])

Parameter	Adjusted Odds ratio (95% CI)	P-value
Female sex	0.93 (0.77–1.12)	0.42
Rural residence	3.16 (2.48–4.02)	0.0001
Risk factor		
No risk factor	Ref.	
Diabetes mellitus	6.43 (5.38–7.69)	0.0001
Renal transplantation	8.61 (4.07–18.20)	0.0001
Others*	1.42 (0.82–2.47)	0.22
Presence of any comorbid illness	0.48 (0.39–0.60)	0.0001
Hypoxia during COVID-19	0.85 (0.66–1.08)	0.18
Diabetic ketoacidosis during COVID-19	3.09 (1.70–5.65)	0.0001
Use of glucocorticoids in the absence of hypoxemia	2.00 (1.37–2.94)	0.0001
Zinc supplementation during COVID-19	2.03 (1.71–2.43)	0.0001
C-reactive protein at admission	1.005 (1.003–1.007)	0.0001
Serum ferritin, µg/L	1.00 (1.00–1.00)	0.79
Neutrophil-to-lymphocyte ratio	1.00 (0.99–1.01)	0.85

*Includes malignancies, hematological malignancies, immunosuppressive therapy, HIV, and others.

Appendix Table 7. Logistic regression analysis showing factors associated with mortality at twelve weeks in subjects with COVID-19-associated mucormycosis (complete case analysis)

Parameter	Adjusted Odds ratio (95% CI)	P-value
Age	1.02 (1.01–1.04)	0.0001
Sex	1.02 (0.76–1.37)	0.92
Risk factor		
No risk factor	Ref.	
Diabetes mellitus	1.30 (0.93–1.82)	0.13
Renal transplantation	2.74 (1.11–6.83)	0.03
Others*	1.31 (0.46–3.76)	0.62
Presence of any comorbid illness	1.34 (0.95–1.88)	0.09
Hypoxemia during COVID-19 illness	1.34 (1.04–1.73)	0.03
Site of involvement by mucormycosis		
Rhino-orbital mucormycosis (ROM)	Ref.	
ROM with brain involvement	2.80 (1.99–3.93)	0.0001
Other sites†	1.51 (0.92–2.46)	0.10
Primary combination medical therapy	0.49 (0.37–0.63)	0.0001
Combined medical and surgical treatment	0.15 (0.10–0.21)	0.0001

*Includes hematological malignancies, immunosuppressive therapy, and HIV infection.
†Includes pulmonary, gastrointestinal, disseminated, and renal mucormycosis.

Appendix Table 8. Summary of the case-control studies assessing factors associated with mucormycosis in COVID-19

Reference	Country	CAM (n)	COVID-19 controls (n)	Exposure assessed	Key findings	Remarks
Muthu/2021 (*1*)	Chandigarh, India	28	29	Zinc	Univariate analysis showed diabetes mellitus was higher in the cases than in the controls. Zinc levels were similar in cases and controls, measured at different time points in cases and controls.	Small sample size, Limited factors assessed. Increased in-vitro growth of *Rhizpus arrhizus* isolates from CAM in zinc-enriched fungal culture medium
Bansal/2022 (*2*)	Gurugram, India	11	50	Demography, HbA1c, serum creatinine, lymphocyte count, D-dimer, ferritin, treatment of acute COVID-19, severity of COVID-19	Univariate analysis suggested higher HbA1c, nadir lymphocyte count, D-dimer, and ferritin in cases than controls	Small sample, included only kidney transplant recipients
Arora/2022 (*3*)	New Delhi, India	152	200	Diabetes mellitus, glucocorticoids, COVID-19 severity, hyperglycemia, nasopharyngeal swabs, zinc therapy, masks	Diabetes mellitus, glucocorticoids, hyperglycemia, a higher number of nasal swabs, and cloth mask usage were predictors on CAM on multivariate analysis	Single center, small sample size
Karat/2022 (*4*)	Bengaluru, India	69	138	Demography, diabetes mellitus, renal function tests, serum ferritin, COVID-19 severity indicators	Random blood sugar, glycated hemoglobin, and elevated C-reactive protein associated with CAM on multivariate analysis	Small sample size, limited factors assessed. Age and sex-matched controls
Kumar/2022 (*5*)	Chandigarh, India	28	26	Diabetes mellitus, glucocorticoids, serum iron, TIBC, serum ferritin	Diabetes mellitus and lower TIBC were predictors of CAM on multivariate analysis	Small sample size, limited factors assessed
Kumar/2022 (*6*)	Wardha, India	55	50	Demography, COVID-19 severity, glucocorticoid, and zinc therapy, diabetes mellitus, complete blood count, renal and liver function tests,	On univariate analysis higher proportion of CAM cases had zinc use	Small sample size, single center, limited factors assessed, unadjusted analysis
Muthu/2022 (*7*)	Chandigarh, India	24	24	Diabetes mellitus, COVID-19 hypoxemia, serum GRP-78 levels	Serum GRP78 levels were significantly higher in CAM than in COVID-19 controls (after adjusting for diabetes and hypoxia) on multivariate analysis	Small sample size, single-center, limited factors assessed
Pandit/2022 (*8*)	New Delhi, India	61	60	Demography, diabetes mellitus, alcohol, smoking, zinc, glucocorticoids, COVID-19 admission parameters	Baseline serum creatinine and d-dimer associated independently with CAM on multivariate analysis	Small sample size, single-center, limited factors assessed
Patel/2022 (*9*)	Ahmedabad, India	64	205	Demography, Diabetes, COVID-19 hypoxia, hospitalization, laboratory, and treatment parameters	Diabetes mellitus, new-onset diabetes, glucocorticoid therapy, home isolation on multivariate analysis	Small sample size, single-center
Ponniah/2022 (*10*)	India; multicenter	383	487	Demography, housing details, occupation, comorbid illness, COVID-19 severity, and treatment. Details of mask and nasal wash during COIVD-19 illness	Hospitalized COVID-19 patients with CAM were associated with diabetes mellitus, glucocorticoid use, and frequent nasal wash on multivariate analysis Non-hospitalized COVID-19 developing CAM were associated with diabetes mellitus, elevated	Separate analysis for hospitalized and non-hospitalized COVID-19 subjects

					plasma glucose, use of cloth mask, and glucocorticoid therapy	
Popli/2022 (*11*)	Rohtak, India	23	23	Vitamin D	Univariate analysis suggested lower vitamin D in cases than in controls	Time of blood collection in cases vs. controls is not clearly mentioned
Vasanthapuram/2022 (*12*)	India; multicenter	179	361	Male sex, diabetes mellitus, glucocorticoid therapy for covid-19, hypoxemia, anti-IL6	Male sex, diabetes mellitus, glucocorticoid therapy for covid-19, and hypoxemia were predictors of CAM on multivariate analysis	Only ROCM, limited factors assessed
Yesupatham/2022 (*13*)	Kolar, India	40	44	D-dimer, ferritin	Univariate analysis suggested higher D-dimer and ferritin in cases than in the controls	Time of blood collection in cases vs. controls is not clearly mentioned

*CAM, COVID-19-associated mucormycosis; GRP-78, 78 kg-Dalton glucose-regulated protein; HbA1c, glycated hemoglobin; ROCM, rhino-orbito-cerebral mucormycosis; TIBC, total iron binding capacity.

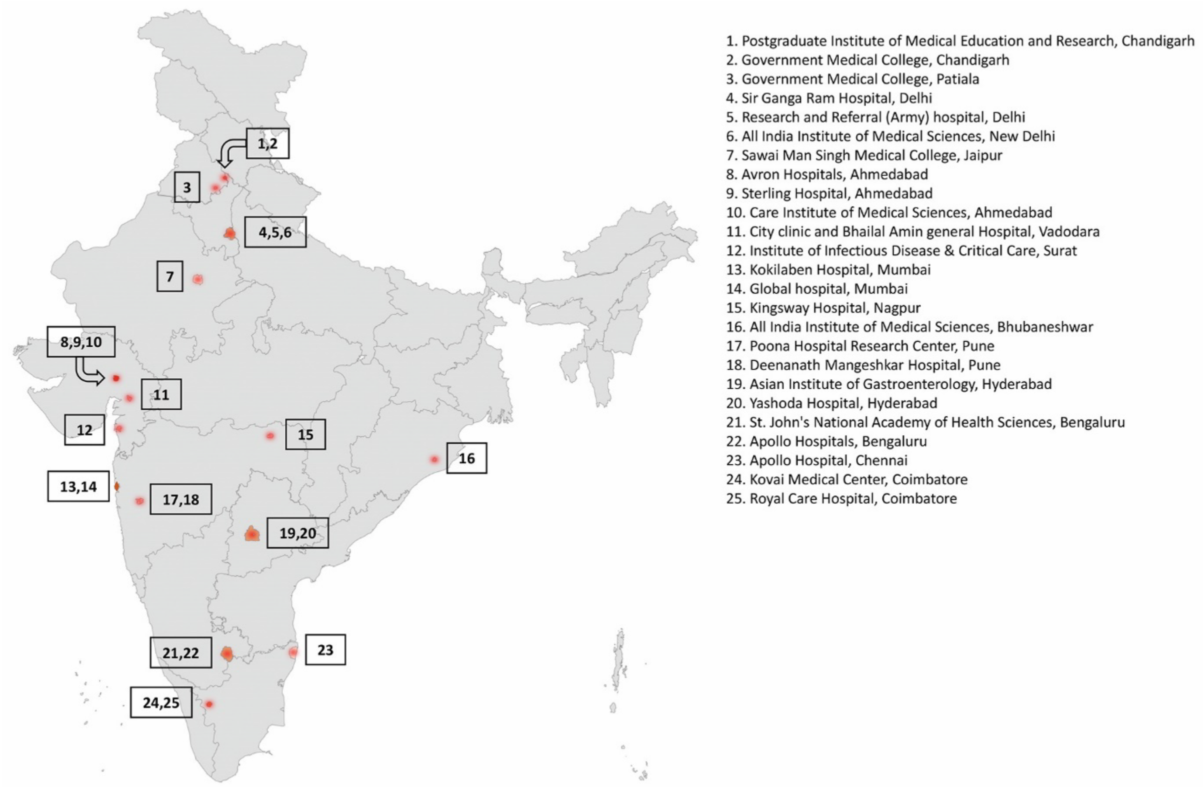

Appendix Figure. Representative map showing the distribution of the various participating centers (numbered 1–25) in multicenter case-control study of COVID-19–associated mucormycosis outbreak, India, January–June 2021
